# Systematic review of health-related quality of life (HRQoL) issues associated with gastric cancer: capturing cross-cultural differences

**DOI:** 10.1007/s10120-022-01309-6

**Published:** 2022-06-11

**Authors:** Alison Rowsell, Samantha C. Sodergren, Vassilios Vassiliou, Anne-Sophie Darlington, Marianne G. Guren, Bilal Alkhaffaf, Chantelle Moorbey, Kristopher Dennis, Mitsumi Terada

**Affiliations:** 1 School of Health Sciences, University of Southampton, Highfield, Southampton, SO17 1BJ UK; 2 Bank of Cyprus Oncology Centre, Nicosia, Cyprus; 3 Department of Oncology, Oslo University Hospital, Oslo, Norway; 4 Faculty of Medicine, Institute of Clinical Medicine, University of Oslo, Oslo, Norway; 5 Department of Oesophago-Gastric & Bariatric Surgery, Salford Royal Hospital, Northern Care Alliance NHS Foundation Trust, Salford, UK; 6 Division of Cancer Sciences, Faculty of Biology, Medicine and Health, School of Medical Sciences, University of Manchester, Manchester, UK; 7 Division of Radiation Oncology, The Ottawa Hospital Cancer Centre, Ottawa, K1H 8L6 Canada; 8 Department of Radiology, University of Ottawa, Ottawa, K1N 6N5 Canada; 9 Asian Partnerships Office, Department of International Clinical Development/International Trials Management Section, Clinical Research Support Office, National Cancer Center Hospital, Tokyo, Japan

**Keywords:** Gastric cancer, Cross-cultural comparison, Health-related quality of life, Patient-reported outcome measures

## Abstract

The treatment landscape for gastric cancer (GC) is constantly evolving with therapies affecting all aspects of health-related quality of life (HRQoL) which need careful monitoring. While there are HRQoL measures designed specifically to capture issues relevant to patients with GC, these might be outdated and only relevant to patients in westernised cultures. This review identifies the patient-reported measures used to assess HRQoL of patients with GC and compares the HRQoL measures used across cultures including East Asia, where GC is more prevalent. We conducted a systematic review of publications between January 2001 and January 2021. A total of 267 papers were identified; the majority (66%) of studies involved patients from East Asian countries. Out of the 24 HRQoL questionnaires captured, the European Organisation for Research and Treatment of Cancer Core Cancer measure (QLQ-C30) was the most widely used (60% of all studies and 62% of those involving patients from East Asian countries), followed by its gastric cancer-specific module (QLQ-STO22, 34% of all studies and 41% from East Asia). Eight questionnaires were developed within East Asian countries and, of the 20 studies including bespoke questions, 16 were from East Asia. There were six qualitative studies. HRQoL issues captured include diarrhoea, constipation, reflux, abdominal pain and abdominal fulness or bloating, difficulty swallowing, restricted eating, and weight loss. Psychosocial issues related to these problems were also assessed. Issues relating to the compatibility of some of the westernised measures within East Asian cultures were highlighted.

## Introduction

Gastric Cancer (GC) is one of the world’s most prevalent and deadliest cancers. In 2018, GLOBOCAN estimated 783,000 deaths attributable to GC [1]. In East Asia: Japan, China, Korea, Mongolia, Taiwan, and Macau, the incidence of GC is higher than in Europe [1, 2]. In 2018, South Korea, Mongolia, Japan, and China constituted the top four countries contributing the highest rates of GC per 100,000 of the population [1] and only one European country, Belarus, figured in the top ten. Due to routine screening for GC, patients from Asia are frequently diagnosed earlier than their European counterparts [2].

The treatment strategy for GC has dramatically changed in the last 10–15 years [2]. Endoscopic or surgical resection is recommended for early tumours [2]. New perioperative treatments have been introduced including the FLOT regimen, 5-fluorouracil, leucovorin, oxaliplatin and docetaxel, with improved survival compared to previous chemotherapy regimens [3]. New targeted agents include anti-angiogenic compounds, trastuzumab combined with platinum and fluoropyrimidine-based chemotherapy for HER-2-positive patients [2], as well as checkpoint inhibitors administered for advanced disease [4, 5]. Contemporary operative treatments using less-invasive approaches have also been introduced, including laparoscopic and robotic surgery. These systemic treatments and surgical advances may have more favourable outcomes in terms of treatment-related toxicities; however, they have unusual side effects previously not seen, including skin rashes, mucositis, and peripheral neuropathy [6, 7] while surgical-related side effects include ‘dumping syndrome’, reactive hypoglycaemia and chronic nutritional problems [8–11].

Treatment-related side effects can have a widespread impact on HRQoL, including physical health, psycho-social well-being, relationships, and independence [12–15]. Complications need to be monitored to implement management strategies, treatment modification or cessation, which is critical from the Healthcare Professional perspective [16, 17]. While the Common Terminology Criteria for Adverse Events (CTCAE) [18] represents the gold standard clinician rated tool for reporting severity of adverse events in clinical trials, patient-reported outcome measures (PROMs) consider HRQoL from the patient’s perspective. Examples of PROMS used in GC include the generic, non-cancer-specific, instruments, such as the Medical Outcomes Survey Short Form survey (SF-36) [19] and the EuroQol group EQ-5D [20] and the cancer-specific measures, such as the Functional Assessment of Cancer Therapy (FACT-G) [21] and the European Organisation for Research and Treatment of Cancer (EORTC) Quality of Life Questionnaire (QLQ) Core 30 (QLQ-C30) [22]. While these measures allow for comparisons across disease and tumour sites, they might overlook the specific and relevant concerns of patients with a particular cancer type such as GC.

Questionnaires designed to capture the HRQoL issues of specific relevance to patients with GC, include the EORTC and FACT gastric cancer-specific modules QLQ-STO22 [23, 24] and the FACT-Ga [25], both designed to supplement their core measures QLQ-C30 and FACT-G. The Gastrointestinal Quality of Life Index (GLQI) [26] and the Post-gastrectomy Syndrome Assessment Scale-45 (PGSAS-45) [27] represent condition specific measures, applicable to gastrectomized patients. These measures are predominantly westernised. The Quality of Life Instruments for Cancer Patients: STomach cancer (QLICP-ST) [28] was developed in response to reported shortcomings of Westernised measures using some items incompatible with Chinese culture, but to date has not been widely applied. Of the Westernised measures, the QLQ-STO22 and FACT-Ga are the most well-known within the field. The QLQ-STO22 includes 22 questions covering five domains: dysphasia; pain/discomfort; dietary restrictions; upper gastro-intestinal symptoms; specific emotional problems; and three single items: dry mouth; body image and hair loss. Although the QLQ-STO22 was developed in westernised cultures, including the UK, France, Germany, and Spain, its use is evolving [29], and it has now been used in multiple trials worldwide and clinically validated to assess the importance of included items in Japan [29, 30], Taiwan (Chinese version) [31], Mexico [32] and Iran [33].

Since GC is more prevalent in East Asia [2], it is important to understand whether the current HRQoL measures applied, like the QLQ-STO22 and FACT-Ga, are relevant to East Asian countries or whether any treatment or culturally specific adaptations or additions are needed. The way in which illness, such as cancer, is perceived and experienced is shaped by cultural and social context [34–36]. In a comparison between HRQoL of patients with breast cancer in the Netherlands and Japan, those from Japan reported better functioning while global quality of life scores were similar. It was suggested that in Japan, the quality of close relationships may contribute more to well-being than in European societies, while for women in western societies, achieving career goals and financial success or career may be more important. A further consideration relates to cross-cultural differences in access to healthcare resources and the economic burden of cancer experienced by patients. These differences need to be taken into account when considering HRQoL assessment across cultures. Studies exploring how HRQoL scales function across groups found differences in understanding [36]. HRQoL measures as well as differences in language assessment and linguistic equivalence including acceptability and interpretation of language [37].

This systematic review forms part of a programme of research addressing whether measures developed within westernised cultures, such as the STO22 cover the issues of relevance and importance to patients with GC in East Asia where GC incidence is highest. The following objectives are addressed in this review: 1. Identify the measures used to assess HRQoL in GC; 2. Examine cross-cultural differences in the assessment and reporting of HRQoL issues; 3. Identify challenges in the application of HRQoL measures across cultures.

## Methods

This systematic review was conducted using the Preferred Reporting Items for Systematic Review and Meta-Analysis (PRISMA) [38] framework.

### Searches and information sources

Searches for publications on patients with GC or esophagogastric Junction Carcinoma (EGJC) within 2 cm of the esophagogastric junction, ≤ 1 year since treatment were carried out. A detailed search of four databases: MEDLINE (Ovid SP), CINAHL Complete (EBSCO host), PsycInfo (EBSCO host) and EMBASE (EBSCO host) was conducted to search for literature published between January 2001 and January 2021. The start period of 2001 was used as a cut-off, based on the date of an earlier systematic review carried out by Vickery and colleagues on behalf of the EORTC Quality of Life Group [23] to inform the development of the QLQ-STO22.

Search expressions were created using the Boolean operator OR between each term in each area and the Boolean operator AND between each area (see Table 1). Care was taken to adapt search expressions for different databases as necessary. The reviewers were also vigilant with respect to identifying new search terms during the review process.

**Table 1:** Search terms used

Area	Terms
Cancer	Cancer Carcinoma Malignancy Neoplasm Oncology Tumor or Tumour
Gastric	Gastric Stomach Gastrectomy
HRQoL	Health-related quality of life HRQoL Quality of life QoL Outcome assessment (health care)
Outcomes/HRQoL outcome measures	HRQoL outcome measures Patient-reported outcome measures

Database searches were supplemented by manually checking references of selected full-text papers. Papers reporting cancers of multiple origins were included if separate data were reported for gastric cancer patients. Quantitative and mixed methods design studies were included as well as those using qualitative methods to capture HRQoL issues or patient-reported symptoms related to GC in any country. Conference proceedings, theses, cost-effectiveness studies and other papers reporting on studies which were not primary studies were excluded. Full details of inclusion criteria are provided in Table 2. All criteria had to be satisfied for a paper to be included.

**Table 2:** Inclusion and exclusion criteria

Inclusion criteria
Papers reporting on HRQoL from the perspective of the patient and excluding clinician rated measures
Papers including patients diagnosed with GC (on or off treatment) and within or equal to 1 year of treatment. This includes patients with Upper gastro-intestinal tumours (stomach only) or patients with cancer within 2 cm of the esophagogastric junction. This will not include patients with upper gastro-intestinal tumours if they are oesophageal, or duodenum tumours or patients with oesophageal cancer not within 2 cm of the EGJC or where location is not specified, or patients with concurrent disease
This will include studies on patients with different diagnoses alongside GC, as long as HRQoL data for people with GC are reported separately
Papers reporting studies on adult patients aged 18 years and above diagnosed with gastric/stomach cancer
Papers reporting studies involving patients from all countries including East Asia (Japan, South Korea, North Korea China, Taiwan, Mongolia, and Macau)
Papers published between January 2001 and January 2021
Papers published in English Language
Studies including the following designs: 1. Randomised controlled trials
2. Trials of quasi-experimental design (observational, case–control)
3. Qualitative studies
4. Mixed methods
Conference proceedings, theses, protocols, cost-effectiveness studies, studies reporting on animals and those not including primary data (e.g., reviews, case studies, expert opinion, theoretical papers, policy documents, guidelines, consensus, letters, editorials) will be excluded
Relevant grey literature from searches

### Paper selection

Database search results were imported into Endnote [X8] and de-duplicated. All references (titles and abstracts) were subsequently transferred to Rayyan QCRI (rayyan.qcri.org), an online systematic review management platform, and a thorough screening process conducted. In stage one, two independent reviewers [AR, SS] reviewed all title and abstracts in Rayyan against the inclusion criteria (Table 2) for full-text review and any discrepancies were resolved. In the event of any remaining doubt, the full paper was obtained. At stage two, all full texts were reviewed by AR and independently double-screened by a second reviewer [SS, MT, MG, KD, CM, BA]. A third reviewer resolved any eligibility disagreements. Inter-rater agreement percentage was reported.

### Data extraction and analysis

A standardised form for data extraction, adapted from the minimum data checklist [39], was created in Microsoft Excel. All extracted data were reviewed by AR and checked by a second reviewer [SS]. Carefully targeted data extraction included: patient group, patient-reported measures used including HRQoL instruments and bespoke questions; geographical location of studies and patient-reported HRQoL issues and symptoms identified in results sections of papers. New issues that were either not covered by existing measures, or that authors reported as novel were also recorded. Measures used to evaluate these issues were recorded. Culturally specific factors were identified by reviewing the narrative provided by authors in the results and discussion sections of papers. Papers reporting studies from East Asia were categorised as those originating from countries based on the World Population review [40] (see Table 2 for the full list).

## Results

**Fig. 1:**
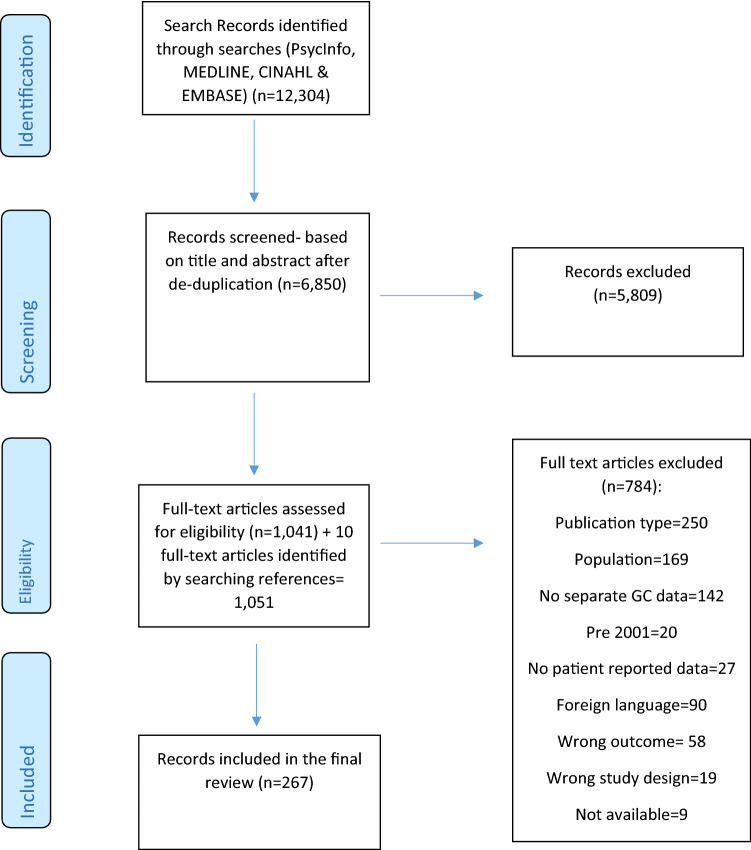
PRISMA diagram of the selection process

The database searches generated 12,304 hits (Fig. 1). During stage 1, 6850 titles and abstracts were screened and 5809 of these were excluded. There was a high level of inter-rater agreement between reviewers (98%; 6752/6850). Screening identified 1041 full-text papers for review and 10 identified through checking full-text paper references. Inter-rater agreement for full-text reviewing was 92.3%. Altogether, 1051 full-text papers were reviewed and 784 were subsequently excluded (of these, nine could not be obtained). The final review included 267 papers; 164 (61%) of these were from East Asia: including 60 from China, 48 Japan, 50 Korea, 3 Taiwan, 1 Japan/Taiwan, 1 Japan/Korea, 1 from China, Japan, and Korea. Eleven papers (4%) reported global studies, including at least one East Asian country.

The remaining papers represented 92 (34%) studies from countries outside of East Asia including: 10 from Germany, 7 Italy, 6 Poland, 5 Sweden, 4 the UK, 3 Spain, France, and Portugal, 2 Turkey, 7 Europe; 10 from Iran, 7 the USA, 4 Canada and India and 2 Colombia. The following countries included only one study: Finland, Vietnam, Singapore, Greece, Switzerland, Lithuania, Serbia, Ireland, Greece, Israel, and Australia. Papers reported mostly trials (127; 48%), e.g., comparing treatment protocols, nutritional supplements or management strategies; cohort studies (62; 23%); prospective studies (38; 14%); and the development and validation of HRQoL measures (9; 3%). Studies also reported on coping strategies, adjustment and acceptance of GC, nutritional status, unmet needs, and educational and self-care interventions. Six papers [23, 41–45], including 5 from non-East Asian countries, reported qualitative studies; three involved interviews with patients about symptoms experienced, such as gastrointestinal problems, fatigue, weight loss, and dietary restrictions, [41–43] and one about treatment preferences [44] whereby maintenance of self-care and minimising toxicities were rated as priorities. Two incorporated qualitative methodologies as part of a questionnaire development process [23, 45].

### Measures used to capture the HRQoL issues of patients with GC

A total of 24 measures were used to assess a wide range of HRQoL concerns in patients with GC (Table 3), including generic (non-cancer-specific) measures (e.g., SF-36, EQ-5D) [19, 20], generic cancer measures (e.g., FACT-G, QLQ-C30,) [21, 22] GC-specific measures (e.g., FACT-Ga, QLQ-STO22) [23–25], measures specific to oesophageal or oesophago-gastric cancer (e.g., EORTC QLQ Oesophago-Gastric (QLQ-OG25), EORTC QLQ Oesophageal (QLQ-OES18) [46, 47]. In addition, measures used include those that can be applied to general gastrointestinal conditions [26, 48, 49] as well as those specific to symptoms and functioning post-gastrectomy [27] or general cancer treatment toxicities, such as nausea, vomiting and fatigue [50–52]. In addition, 20 studies (e.g., [53, 54] applied their own bespoke questions or measures often as a supplement to validated measures to capture additional treatment-related symptoms or psycho-social issues.

**Table 3:** Application of measures by region of the world

Measures	Areas (domains/items assessed)	Countries where measures are used^a^		
		East Asia	Non-East Asian countries	East Asia and non-East Asian countries combined
Generic				
EuroQol-5 dimension (EQ-5D) [20]	Mobility, Self-care, Pain, Usual activities, and Psychological status	12	5	17
Medical Outcomes Study Short-form (SF36/SF-36) [19]	Physical and Social functioning, Physical and Emotional role limitations, Mental health, Energy, Pain, and General health perceptions Domains Physical and Mental component summaries	2	1	3
Spitzer Quality of Life Index [60]	Activity, Daily living, Health, Support of family and friends, Outlook	5	0	5
15D (15-dimensional measure of HRQoL) [61]	Breathing, Mental function, Speech, Vison, Mobility, Usual activities, Vitality, Hearing, Eating, Elimination, Sleeping, Distress, Discomfort and symptoms, Sexual activity, Depression	1	0	1
World Health Organisation—Quality of Life WHOQOL Scale (also including WHOQOL-BREF) [62]	Physical health, Psychological health, Social relationships, Environmental health,	1	3	4
Generic cancer				
European Organization for Research and Treatment of Cancer (EORTC) Quality of Life Questionnaire-Core 30 (QLQ-C30) [22]	Domains: Physical functioning, Emotional functioning, Social functioning, Role functioning, Cognitive functioning. Items: Fatigue, Pain, Nausea/Vomiting, Constipation, Diarrhoea, Insomnia, Dyspnoea, Appetite loss, Financial Difficulties, Global health, Global quality of life	108	51	159
Functional Assessment of Cancer Therapy—General (FACT-G) [21]	Physical well-being, Social/Family well-being, Emotional well-being	2	3	5
Functional Assessment of Cancer Therapy: Fatigue (FACT-F) [50]	Fatigue	0	1	1
Quality of Life Questionnaire for Cancer Patients treated with anticancer drugs (QOL ACD) [27]	Daily Activities, Physical condition, Social activities, Mental status, Psychological status			
Functional Living Index-Emesis (FLIE) [51]	Nausea Vomiting	2	0	2
Gastric Cancer-specific				
European Organization for Research and Treatment of Cancer (EORTC) Quality of Life Questionnaire—Gastric Cancer (QLQ-STO22) [23, 24]	Domains: Dysphagia, Dietary restriction, Pain, Reflux, Anxiety Items: Disease symptoms, treatment side effects, Dry mouth, Body image, taste problems	72	18	90
Functional Assessment of Cancer Therapy—Gastric Cancer (FACT-Ga) [25]	Gastric cancer subscale items: Weight loss, Appetite loss, Reflux, Dietary restrictions, Pain/discomfort when eating, Feeling full/heavy in the stomach, Stomach swelling/cramps, Dysphagia, Change in eating habits, Enjoyment of meals (socially), Interference of usual activities, Avoidance of going out, Worry over stomach problems, Discomfort/pain in stomach area, Flatulence, Diarrhoea, Fatigue, Weakness, Plans for the future	3	4	7
GC-PROM: Patient-reported outcome measure for Chinese patients with gastric cancer [55]	Physical: Abdominal symptoms, Systemic symptoms, Physical state, Independence; Psychological: Anxiety, Depressed, Pessimism; Social: Social support, Social adaptation; Therapeutic: Effectiveness, Satisfaction, Compliance, Drug side effects	1	0	1
KOQUSS-40: Symptom-Focused Quality of Life Questionnaire for Gastric Cancer Patients after Gastrectomy [45]	General quality of life, Indigestion, Dysphagia, Reflux, Dumping syndrome, Bowel habit change, Constipation, Psychological factors, Worry about cancer, Scar problems, Financial problems	1	0	0
Korenaga’s Score Scale/Chew wun Wu special symptom score [56, 57]	Appetite, Consistency of digested food, Volume of digested food, Frequency of eating, Eating time, Postprandial abdominal fullness, Heartburn, Diarrhea, and Constipation persistence, Insomnia, Body weight changes, Swallowing problems, Vomiting, Dizziness	2	1	3
Post-gastrectomy Syndrome Assessment Scale-45/037 (PGSAS45/PGSAS37) [27]	In addition to items from the SF-8 and GSRS: PGSAS-specific items: Bile regurgitation, sense of food sticking, Postprandial fullness, Early satiation, Lower abdominal pains, Dumping (early and late) symptoms. Dietary intake (amount and quality of ingestion), Work, Satisfaction with daily life	2	2	4
System of Quality of Life Instruments for Cancer Patients: Stomach Cancer (QLICP-ST) [28]	Physical, Psychological, Common symptoms and side effects	1	0	1
Quality of life assessment based on Traditional Chinese Medicine for advanced gastric cancer (QLASTCM-Ga) [58]	Unity of the body and spirit Correspondence between man and universe Specific module General module	1	0	1
Oesophageal-specific				
European Organization for Research and Treatment of Cancer (EORTC) Quality of Life Questionnaire—Oesophago-Gastric Modules (OG25) [46]	Domains: Dysphagia, Eating restrictions, Reflux, Odynophagia, Pain, Anxiety	3	2	5
European Organization for Research and Treatment of Cancer (EORTC) Quality of Life Questionnaire – Oesophageal Cancer (OES18) [47]	Domains: Dysphagia, Eating, Reflux, Pain. Items: Saliva swallowing, Choking, Dry mouth, Taste, Coughing, Talking	3	1	4
Gastrointestinal-specific				
Gastrointestinal Quality of Life Index (GLQI) [26]	Gastrointestinal symptoms, Emotion, Physical function, Social function, Medical treatment	1	5	6
Gastrointestinal Symptom Rating Scale (GSRS) [48]	Reflux, Abdominal pain, Indigestion, Diarrhoea, Constipation	5	1	6
Patient Assessment of Gastrointestinal Disorders-Symptoms Severity Index (PAGI-SYM) [49]	Heartburn/regurgitation, Nausea/vomiting, Fullness/early satiety, Bloating, Upper abdominal pain, Lower abdominal pain	1	0	1
Gastroesophageal reflux disease (GERD)-QOL Questionnaires [59]	Daily activity, Diet, Psychological well-being	1	1	2
Bespoke questions developed for the purpose of the study	Post-gastrectomy symptoms Dumping syndrome symptoms Satisfaction Psychological functioning Social functioning	16	4	20

^a^Studies often use more than one measure

The most widely used measure was the generic cancer QLQ-C30 [22] used in 159 (60%) studies, either as a standalone or supplemented with the GC-specific QLQ-STO22 in 90 (33%) studies. The generic, non-cancer-specific EQ-5D [20] was the next most used validated measure, however this was only used in 6% of studies. Other generic cancer and GC-specific measures were also used sparingly globally, including the FACT-Ga [21] used across 7 studies and two gastrointestinal measures: the Gastrointestinal Quality of Life Index (GLQI) [48] and the Gastrointestinal Symptom Rating Scale (GSRS) [27] used in six studies each.

In addition to bespoke questions added to match the researchers’ areas of interest, eight measures [27, 28, 45, 54–58], were developed in East Asia, and the PGAS45 [27] includes clinically important items selected by the Japan Post-gastrectomy Syndrome Working Party. Across all measures, the most widely assessed construct pertains to physical symptoms and this is particularly true for the GC or gastrointestinal specific measures which focus on gastrointestinal symptoms of disease and treatment such as gastrectomy. General physical symptoms assessed (e.g., by the EQ5D, QLQ-C30, FACT-G) [5, 21, 22] which are not necessarily cancer or GC-specific include mobility, nausea, vomiting, appetite and weight loss, diarrhoea, constipation fatigue, energy, sleeping problems, and pain. GC-specific symptoms assessed (e.g., by the FACT-Ga, QLQ-STO22, and the Symptom-Focused Quality of Life Questionnaire for Gastric Cancer Patients after Gastrectomy (KOQUSS-40)) [23–25] include reflux, difficulty swallowing (dysphagia), dry mouth, eating and dietary restrictions, taste, abdominal pain and fullness, and dumping syndrome, with the PGSAS45 [27] distinguishing between early and late symptoms. The impact of symptoms on functioning is also assessed and, in particular, captured by the generic measures (e.g., by the EQ5D, SF-36, Spitzer Quality of Life Index, the 15D, QLQ-C30 and FACT-G) [19–22, 60, 61].

Psychological or emotional aspects of HRQoL, such as depression (15D, QLQ-C30, Patient-reported outcome measure for Chinese patients with gastric cancer (GC-PROM)) [22, 55, 61] worry and anxiety (QLQ-STO22, GC-PROM, OG25, Symptom-Focused Quality of Life Questionnaire for Gastric Cancer Patients after Gastrectomy (KOQUSS-40)) [23, 24, 31, 45] and distress (15D) [61], are also measured. The social domain covers social or family well-being (SF-36, World Health Organisation Quality of Life Scale (WHOQOL/WHOQOL-BREF), FACT-G,QLQ-C30) [19, 21, 22, 62], ability to go out and engage in social activities, social support (GC-PROM, Spitzer Quality of Life Index) [55, 60], and social enjoyment of meals [25]. Other HRQoL issues captured include sexual functioning (15D) [61], cognitive functioning (QLQ-C30) [22], financial difficulties (QLQ-C30, KOQUSS-40) [22, 45] body image (QLQ-STO22) [23, 24], outlook on life (Spitzer Quality of Life Index) [60], plans for the future (FACT-Ga) [25], and attitudes towards treatment (GC-PROM) [55]. One measure focused on HRQoL in relation to traditional Chinese Medicine [58].

#### Cross-cultural differences in the assessment and reporting of HRQoL issues

As mentioned above, the EORTC measures (QLQ-C30 and QLQ-STO22) were the most frequently used across all studies and this was also the case for studies conducted within East Asian countries. The QLQ-C30 was the measure of choice in 62% of studies involving patients from East Asia and the QLQ-STO22 in 41% of studies in East Asia. Of the eight measures developed within East Asian cultures, only the Korenaga Scale score/Wu’s adapted special score [56, 57] and the GERD QOL Scale [59] have been applied in studies involving patients outside of East Asia. In addition, of the 20 studies including bespoke questions or scales, 16 (80%) were conducted within East Asia.

#### Challenges to the assessment of HRQoL in GC across cultures

Cultural influences on HRQoL assessment were reported in terms of differences across cultures in treatment protocols, health disparities, access to health care resources, cultural views and practices, environmental and psycho-social issues, and linguistic equivalence. Potential environmental factors reported to impact HRQoL included pollution [63] and economic burden, especially for Chinese patients [35].

There were poignant cultural differences even surrounding the conceptualisation of cancer. In some East Asian studies cancer and malignancy were not culturally acceptable topics for discussion [32] and there was a social stigma linked to GC. Lee and colleagues [56, 64] compared HRQoL of Canadian Western and Korean patients and reported lower HRQoL in Korean patients. They suggested that as group participation is an integral part of Korean culture and cancer patients have been known to have to leave their jobs and miss group activities because of their cancer, this might be a significant impact for patients from Korea. Discussion of family and practical problems, spirituality, religion, and sexuality were reported as culturally sensitive and potentially upsetting for some communities [34, 35]. As part of a validation study of the FACT-Ga in Japan [65], the proportion of missing responses to the sex life question “I am satisfied with my sex life”, was highlighted with only 40.5% of respondents answering the question.

Several validation studies exploring the design and content validity of the QLQ-C30 and QLQ-STO22 promoted their validity for use in East Asian culture [30, 31, 66]. Whilst the overall validity of the most widely used GC-specific measures was confirmed, there were reported issues around linguistic equivalence in terms of acceptability and interpretation of language. For example, Huang and colleagues [31] suggested that the QLQ-C30 and QLQ-STO22 measures held the potential to positively impact understanding of well-being in patients from Taiwan, but that questions in the eating restriction scale, may hold different meaning for Taiwanese patients. Validation studies in Mexico [32] and Iran [33], also noted favourable outcomes on most scales of the QLQ-STO22, other than low internal consistency for “have you had trouble enjoying your meals?”. It was noted [33] that patients understood the translation of the question about enjoyment of meals but suggested that it might mean something different in Persian culture compared to in Western cultures. Restrictions to eating or meal-related issues, were widely documented in the literature and there were occasional adaptations to measures linked to East Asian food and culture. For example, Morita et al.’s validation study of the QLQ-STO22 [30], suggested validity for use in Japan, however, they recommended that the question referring to a ‘bloated feeling’ should be replaced with one referring to ‘retaining gas’. Furthermore, they speculated that problems linked to dysphagia and early satiety, might be linked to a staple Japanese food, rice, as patients might experience problems enjoying meals because of the need to eat a liquidised form of rice. Garland and colleagues [25] reported that patients found it difficult when questions included the word “stomach” if they had experienced a total gastrectomy and thus removed questions including this word from the Asian version of the FACT-Ga. Reference to cultural practices including traditional Chinese Medicine (including acupuncture, Tai Chi and herbal medicines) and its link to improved HRQoL was a noteworthy feature of several East Asian studies, principally from China [58, 67, 68]. Quan et al. [58] explored HRQoL issues for GC patients by implementing their HRQoL measure for traditional Chinese Medicine for advanced GC to measure the benefits of Chinese medicines for chemotherapy or surgical symptoms. The merits of a measure developed to capture the nuances of Chinese culture, the QLIP-ST [28] were highlighted in a comparison with the QLQ-STO22 [23, 24] in terms of enhanced compatibility with Chinese cultures, clearer structure, greater precision, and ease of interpretation [28].

## Discussion

Our review captures the measures used to identify the full range of HRQoL issues faced by people with GC. The review generated 267 studies using patient-reported HRQoL measures with patients with GC who had recently finished treatment (within a year). Two thirds of these studies were from East Asia, which given the higher prevalence of GC in this part of the world is unsurprising [1, 2]. Measures developed with patients and health care professionals from European countries (QLQ-C30 and QLQ-STO22) were the most widely used and thus the patient-reported HRQoL issues reported in the studies are largely a function of the items included in these measures. Overall, studies conducted in East Asia applied more GC-specific measures, mainly using the QLQ-STO22, compared with studies conducted in countries outside of East Asia. This might again be accounted for by the high and emerging incidence and interest in GC treatment in East Asia, evidenced by the fact that 88% of studies in East Asia identified in this review were conducted since 2010, with the vast majority conducted post-2015. Our findings suggest that the measure of choice does not vary between countries and while the validity of the westernised measures has been queried in some papers (e.g., [28, 55, 64]), evidence suggests that these measures were widely accepted [30, 33]. In non-westernised countries, some content validity issues were noted especially around linguistic equivalence and problems in translating questions in the ‘eating restrictions’ [30, 31, 33]. Questions in these domains may need reassessing in countries outside of East Asia. Eight measures were developed for patients within East Asia and 16 of the 20 studies including bespoke measures were from East Asia suggesting that the existing and widely used westernised measures might have important omissions, such as symptoms relating to dumping syndrome or need adapting to suit East Asian culture.

In addition, the advent of new regimens [3–5] and surgical treatments [8–11] and specific side effects related to these (such as skin rashes, peripheral neuropathy, mucositis), may not be present in the existing HRQoL measures.

Several issues were only reported through ad hoc measures or in interview studies, including changing emotions surrounding food [41] and family communication issues [53]. These studies are particularly informative for providing additional depth, as patients have the freedom to talk about issues of concern to them, rather than responding to a set of pre-defined issues in a questionnaire.

Sexuality and sexual activity were rarely explored in the literature, irrespective of geographic region. Again, this might be a function of these issues not being asked about in the most widely used measures, such as QLQ-C30 and QLQ-STO22. Another possible explanation might link to the cultural unacceptability of discussing sexuality and sexual activity as a topic, particularly in East Asia and the difficulties in applying the sexual well-being scale of the FACT-Ga noted by Maeda and colleagues [65]. More research is needed to explore how acceptable patients with GC find it to be asked about the impact of their cancer on sexuality and whether there are geographical variations. Other overlooked issues included spiritual and religious topics and family communication, and, as with sexual functioning, there are potential cultural differences around acceptability about discussing these issues with less acceptability in some East Asian cultures. Further research is needed to quantify whether there are differences between non-Westernised and Western countries, surrounding these topics.

The impact of traditional Chinese Medicine on HRQoL was an expected feature of the East Asian literature given the high regard placed on traditional practices and one measure specific to capturing the HRQoL issues based on traditional Chinese Medicine [58] was identified in this review. There were also issues relating to language used with the perceived unacceptable use of the word stomach resulting in its removal from the Asian FACT-Ga [25]. Interestingly, the word stomach is also referred to in the widely used QLQ-STO22, but no studies reported this as problematic, although this may be because critiques of measures are rare. Similarly, preferences for using phrases such as ‘retaining gas’, rather than a ‘bloated feeling’ were occasionally voiced by patients [30]. Further research is needed to explore the cultural acceptability of HRQoL measures, and linguistic equivalence issues.

### Limitations

During the selection process, 90 non-English language papers were excluded and many more were automatically excluded as a function of the filter option of database searches, thus we might have overlooked some pertinent issues and measures reported in studies in East Asia. Although we provide an overview of the measures used and thus HRQoL issues reported by patients with GC, our overview of issues is limited by the questions included in the measures; there were only six qualitative studies which offer additional insight into the HRQoL experience of patients. In addition, comparisons in the HRQoL of patients across cultures are limited; we were in a position to compare HRQoL measure usage across cultures rather than differences in the incidence of HRQoL concerns according to culture. This review is also limited by its descriptive focus, and the heterogenous nature of the studies, in terms of methods and measures used and focus and context of the studies. This makes it difficult to make comparisons between studies and ensure issues have not been missed. We have however provided a comprehensive overview of the measures used to assess patient-reported outcomes in GC patients from East Asian countries and countries outside of East Asia but given emerging treatments and new or adapted patient-reported HRQoL measures, we recognise the need for an on-going evaluation of patient-reported issues.

## Conclusion

To our knowledge, this review is the first to provide a comprehensive overview and comparison between East Asia and non-East Asia in HRQoL measures used for GC. Measures provide extensive coverage of HRQoL issues covering issues across physical, social, and psychological domains. The EORTC measures (QLQ-C30 and QLQ-STO22) are the most widely used irrespective of geographical location. While these measures benefit from extensive testing as part of their validation, they were not developed and tested with people from East Asia. Measures developed within East Asian cultures and bespoke questions and instruments offer improved sensitivity to the nuances of East Asian cultural and social contexts and allow for further exploration of psycho-social issues as well as symptoms relating to dumping syndrome or novel treatments. Further research is needed on the cultural relevance of HRQoL measures as well as further consideration of how we might make measures more culturally adaptable and acceptable.
