# Correction to: Systematic review of health-related quality of life (HRQoL) issues associated with gastric cancer: capturing cross-cultural differences

**DOI:** 10.1007/s10120-022-01346-1

**Published:** 2022-10-21

**Authors:** Alison Rowsell, Samantha C. Sodergren, Vassilios Vassiliou, Anne-Sophie Darlington, Marianne G. Guren, Bilal Alkhaffaf, Chantelle Moorbey, Kristopher Dennis, Mitsumi Terada

**Affiliations:** 1 School of Health Sciences, University of Southampton, Highfield, Southampton, SO17 1BJ UK; 2 Bank of Cyprus Oncology Centre, Nicosia, Cyprus; 3 Department of Oncology, Oslo University Hospital, Oslo, Norway; 4 Faculty of Medicine, Institute of Clinical Medicine, University of Oslo, Oslo, Norway; 5 Department of Oesophago-Gastric & Bariatric Surgery, Salford Royal Hospital, Northern Care Alliance NHS Foundation Trust, Salford, UK; 6 Division of Cancer Sciences, Faculty of Biology, Medicine and Health, School of Medical Sciences, University of Manchester, Manchester, UK; 7 Division of Radiation Oncology, The Ottawa Hospital Cancer Centre, Ottawa, K1H 8L6 Canada; 8 Department of Radiology, University of Ottawa, Ottawa, K1N 6N5 Canada; 9 Asian Partnerships Office, Department of International Clinical Development/International Trials Management Section, Clinical Research Support Office, National Cancer Center Hospital, Tokyo, Japan

## Correction to: Gastric Cancer (2022) 25:665–677 10.1007/s10120-022-01309-6

In the original publication of the article, the last author should be “Mitsumi Terada” and not “Mistumi Terada”.

The original article has been corrected.

